# Complete genome sequence of *Pseudomonas* sp. koa227, an anti-oomycete endophytic isolate from the shoot xylem of the Japanese indigenous wine grape *Vitis* sp. cv. Koshu

**DOI:** 10.1128/mra.00971-26

**Published:** 2026-10-14

**Authors:** Toshiyuki Kano, Yoshinao Aoki, Takayuki Asada, Masutoshi Nojiri, Shunji Suzuki

**Affiliations:** 1 Laboratory of Fruit Genetic Engineering, The Institute of Enology and Viticulture, University of Yamanashi 38146, Kofu, Yamanashi, Japan; 2 Agri-Bio Research Center, Kaneka Corporation, Iwata, Shizuoka, Japan; USDA-ARS San Joaquin Valley Agricultural Sciences Center, Parlier, California, USA

**Keywords:** endophyte, *Pseudomonas*, wine grape

## Abstract

Endophytic bacteria residing within grapevines represent a largely unexplored research frontier, highlighting the need to elucidate the physiological properties of individual strains. Here, we introduce a genome sequence of *Pseudomonas* sp. koa227, an anti-oomycete endophytic isolate from the shoot xylem of a Japanese indigenous wine grape.

## ANNOUNCEMENT

The use of endophytic microorganisms is a promising strategy for biocontrol because they can stably colonize host plants without causing disease (1). Among these microorganisms, *Pseudomonas* species contribute to disease suppression by employing a range of mechanisms, including nutrient competition, antimicrobial metabolite production, type VI secretion system, and induced systemic resistance (2, 3).

Shoots of the 35-year-old Japanese indigenous wine grape *Vitis* sp. cv. Koshu, cultivated in the experimental vineyard of the University of Yamanashi, Yamanashi, Japan, were collected on 9 December 2019. Endophytic bacteria were isolated from the shoot xylem following the method described by Hamaoka and Suzuki (4). Among 387 bacterial isolates, koa227 was selected for its antagonistic activity against phytopathogenic oomycete *Plasmopara viticola*, and its genome was sequenced to investigate the underlying mechanisms.

Sequencing and genome assembly were performed by Bioengineering Lab. Co., Ltd. Briefly, genomic DNA was extracted from koa227 cultured in soybean casein digest (Fujifilm Wako, Osaka, Japan) medium at 37°C for 1 day using a Genomic-tip 20G (Qiagen) and sheared using a Megaruptor 3 (Diagenode) to obtain fragments of approximately 7–15 kb. The library was prepared using the SMRTbell Prep Kit 3.0 (PacBio) in combination with the Twist Universal Adapter System (Twist Bioscience). PCR products were verified by electrophoresis using a 5200 Fragment Analyzer System (Agilent Technologies). The polymerase complex was then assembled with the Revio Polymerase Kit (PacBio), and sequencing was conducted on the Revio platform (PacBio). HiFi reads were generated using SMRT Link (version 25.2.0.266456), which removed overhanging adapter sequences to form subreads. The subreads were then aligned to generate circular consensus sequences (CCSs), and CCS reads with an average quality score below 20 were discarded to obtain the final HiFi reads. For filtering, adapter sequences were removed from HiFi reads using lima (version 2.12.0), followed by the removal of PCR-generated duplicates using pbmarkdup (version 1.0.3) (5). Filtlong (version 0.2.1) (6) was then applied to exclude reads shorter than 1,000 bases or with quality scores in the lowest 10% of all reads, and 30,988 HiFi reads (total, 209,546,140 bp; average, 6,762.2 bp; and N50, 7,147 bp) were obtained. HiFi reads of 1,000 bases or longer (26,892 reads) were assembled using Flye (version 2.9.3-b1797) (7) under default settings. The integrity of the assembled genomes was assessed using CheckM2 (version 1.0.1) (8).

The genome consists of three contigs with sizes of 5,909,907 bp (circular chromosome), 53,230 bp (pKoa227-1), and 111,973 bp (pKoa227-2) with 35× coverage. The N50 and the longest contig size are the same (5,909,907 bp). The GC content is 60.5%. CheckM2 indicated low contamination (0.12%) and 100% completeness of the genome. The annotation by DFAST (version 1.6.0) predicted 5,378 coding sequences, 16 rRNA genes, and 65 tRNA genes. While 16S rRNA gene analysis using 9F and 1510R primers placed koa227 close to *Pseudomonas graminis* (Y11150), whole-genome ANI analysis using OrthoANIu version 1.2 (9) further supported its distinct taxonomic status. The highest ANI value was 92.17% with *P. graminis* (CP053746), which is well below the generally accepted species threshold of 95%–96% (10).

## Data Availability

This project was deposited in DDBJ/ENA/GenBank under accession numbers PRJDB37638 (BioProject), SAMD01688595 (BioSample), AP044700 (genome), AP044701 (pKoa227-1), AP044702 (pKoa227-2), and DRR1086542 (Sequence Read Archive RUN).
